# Adult Traumatic Atlantoaxial Rotatory Fixation: A Case Report

**DOI:** 10.1155/2014/593621

**Published:** 2014-03-04

**Authors:** Zaw Min Han, Nobuto Nagao, Toshihiko Sakakibara, Koji Akeda, Takao Matsubara, Akihiro Sudo, Yuichi Kasai

**Affiliations:** ^1^Department of Spinal Surgery and Medical Engineering, Mie University Graduate School of Medicine, 2-174 Edobashi, Tsu, Mie 514-8507, Japan; ^2^Department of Orthopaedic Surgery, University of Medicine, Mandalay, Myanmar; ^3^Department of Orthopaedic Surgery, Mie University Graduate School of Medicine, Japan

## Abstract

We presented a very rare case of adult Fielding type I atlantoaxial rotatory fixation (AARF). We performed awake manual reduction of the dislocation without need for anesthesia, achieving excellent outcomes, and no previous reports have described awake reduction without the need for anesthesia. AARF in this case was attributed to excessive extension and rotation forces applied to the cervical spine. For the management of adult Fielding type I AARF, early diagnosis and early reduction may lead to excellent outcomes.

## 1. Introduction

We treated a very rare adult traumatic atlantoaxial rotatory fixation (AARF) with manual reduction while patient was awake, obtaining excellent outcomes. This case is reported together with a discussion of characteristics in adult cases.

## 2. Case Report

The patient was a 22-year-old man. While driving, he steered in the wrong direction at an intersection and collided with an oncoming car. After complaining of intense cervical pain, he was brought by ambulance to our emergency outpatient unit. He had no previous history of note.

**Figure 1:**
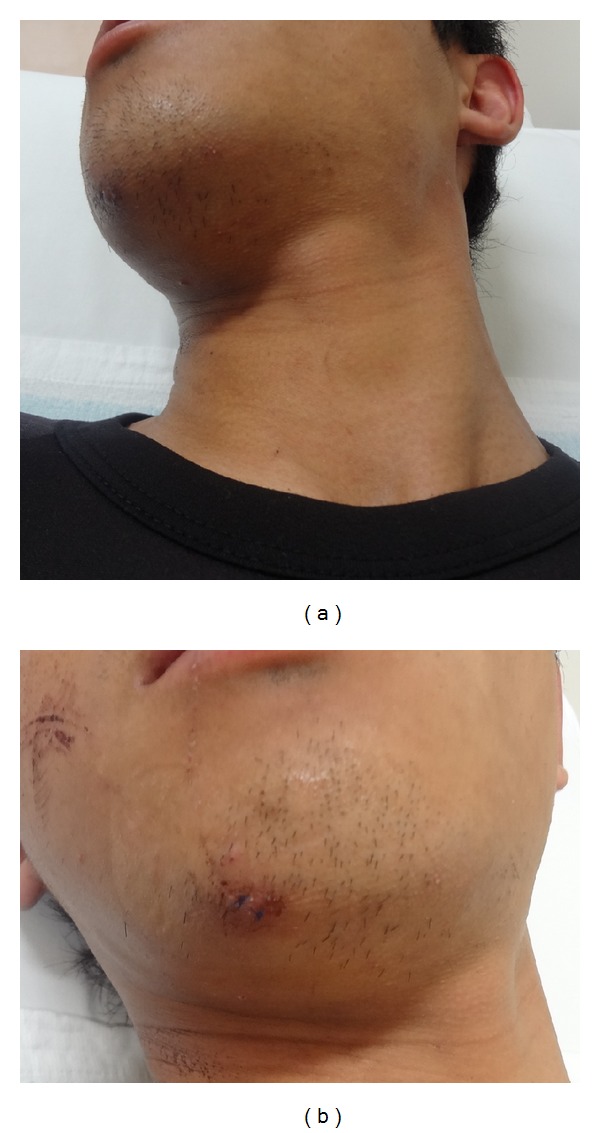
The patient could not move his head at all from its position facing to the right (a). A lacerated wound over the right inferior mandible (b).

**Figure 2:**
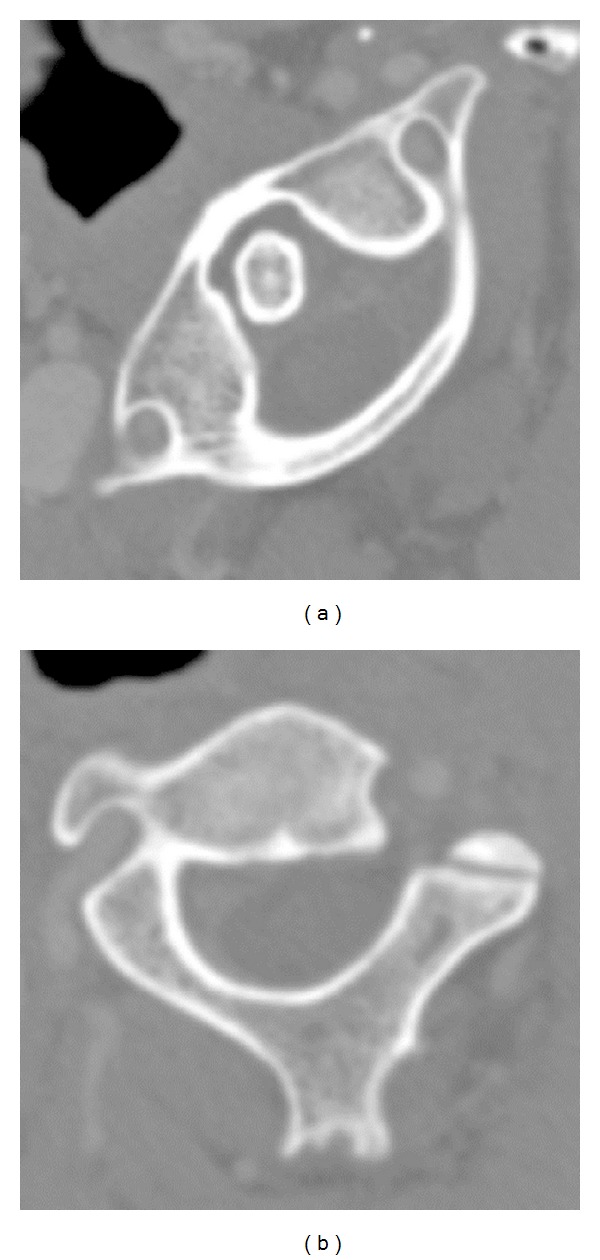
Computed tomography revealed that the atlas was rotated to the right centering on the dens of the axis. (a) Axial view of atlas and (b) axial view of axis.

**Figure 3:**
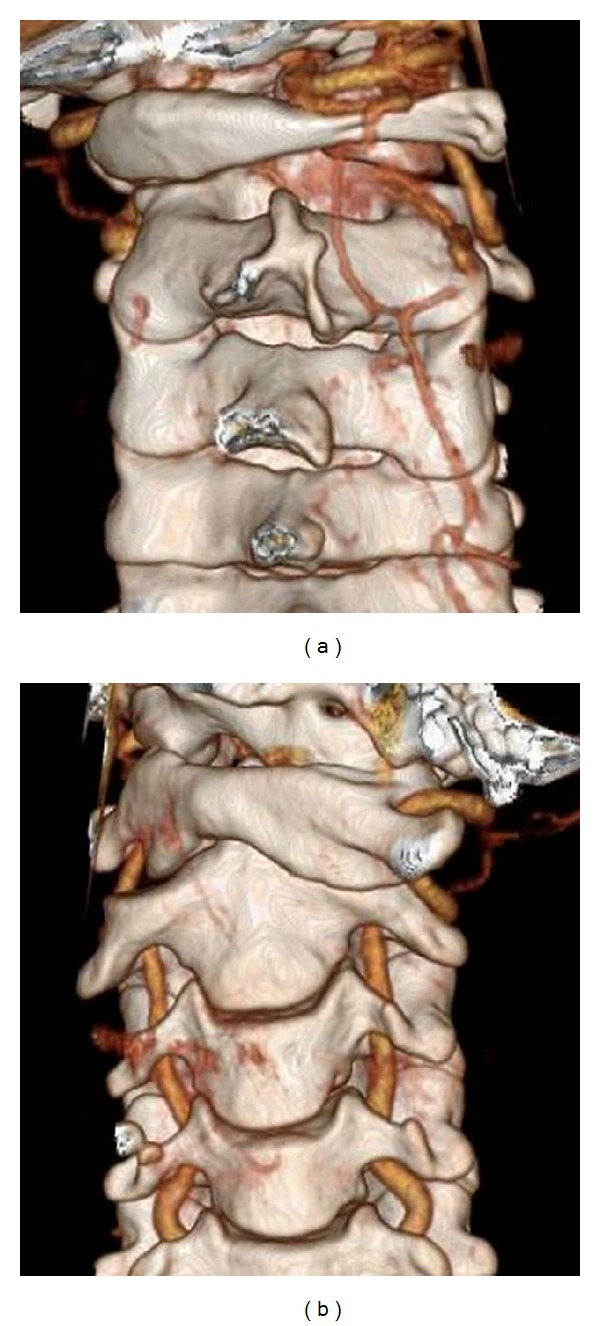
The three-dimensional CT showed atlantoaxial rotatory fixation of Fielding classification type I. (a) Posterior view and (b) anterior view.

On initial consultation, the patient could not move his head at all from its position facing to the right (Figure 1(a)). He had clear consciousness and no motor paralysis or sensory disturbance. A lacerated wound (Figure 1(b)), about 1 cm in length, was observed over the right inferior mandible. Computed tomography (Figure 2) revealed that the atlas was rotated to the right centering on the dens of the axis. However, no findings suggested congenital dysplasia, and no fractures such as the articular process were observed. Based on a three-dimensional CT (Figure 3), we diagnosed AARF of Fielding classification type I [1] without a protruding eccentric jaw position.

Treatment comprised manual reduction while patient was awake about 2 hours after injury. At first, we held the mandible, providing traction in the cephalic direction, confirming that the patient did not develop pain or palsy in the upper extremities, and rotated it slowly to the left to obtain a feeling of reduction. Immediately after reduction, cervical pain was alleviated and no neurological complications were observed.

**Figure 4:**
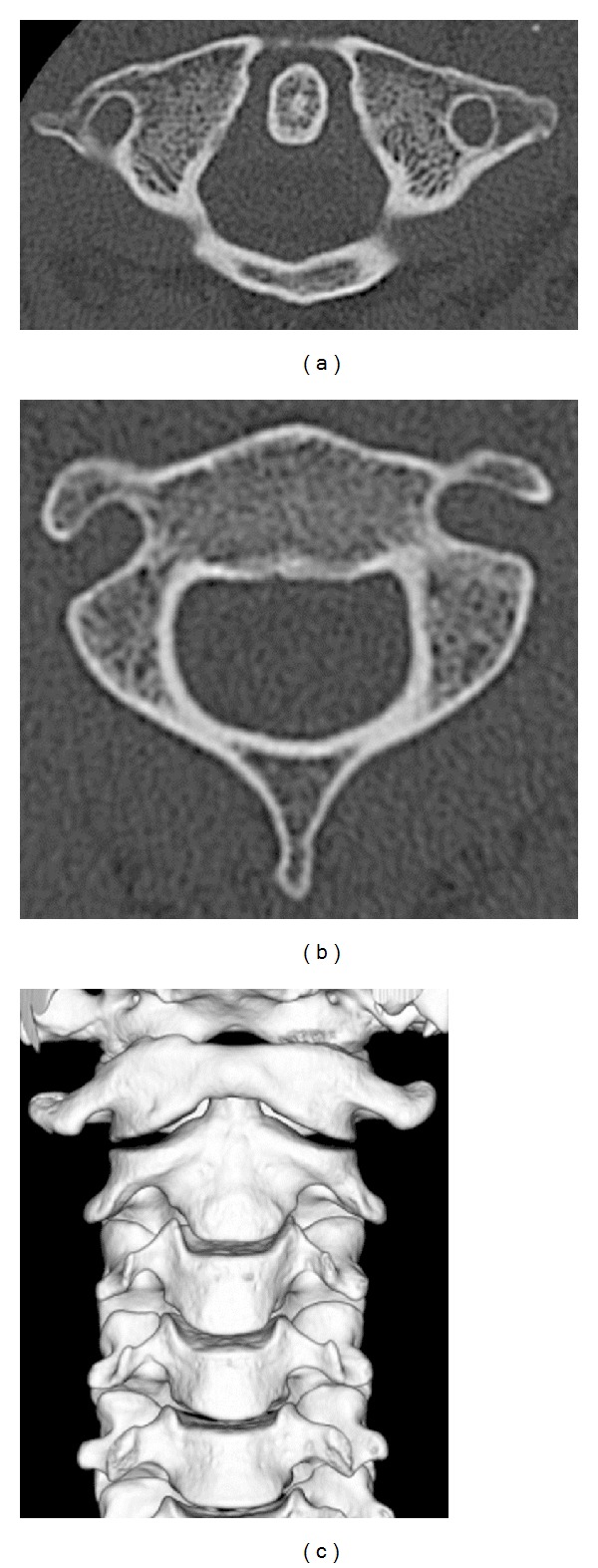
CT after reduction of atlantoaxial rotatory fixation. (a) Axial view of atlas, (b) axial view of axis, and (c) anterior view of three-dimensional CT.

MRI after reduction indicated no major soft tissue damages, and CT after reduction (Figure 4) showed that the dislocation had been reduced, so fixation with a Philadelphia collar was performed. Radiography at 1 month after injury showed no intervertebral instability between C1 and C2, and the collar was removed at that time based on the strong request of the patient. CT at 1 year after injury showed no redislocation, and the patient had no cervical pain and was progressing satisfactorily.

## 3. Discussion

AARF is defined as torticollis caused by atlantoaxial dislocation or subluxation. Causes may include damage to soft tissues such as the articular capsule, transverse ligament, or alar ligaments between C1 and C2 or secondary contracture of the articular capsule or ligament tissues between C1 and C2 facet. This pathology is reported to occur frequently in children, triggered by slight trauma or upper respiratory infection in many cases [2]. The reasons for this predominance in children include (1) the relatively large size of the head in proportion to the rest of the body; (2) insufficient development of the muscular tissues around the neck; (3) increased elasticity of the C1-C2 joint capsule and large rotation angle; and (4) the horizontal configuration of articular facets [3].

Traumatic adult AARF as in the present case is reported very rarely, with only 14 cases identified in the literature since 2000 [4–14]. These reports are summarized in Table 1 and involved 5 men and 9 women between 20 and 52 years old (mean age, 30.3 years). AARF is frequently caused by high-energy trauma, such as that sustained in traffic accidents or falls. Since it is caused by high-energy trauma, patients often present with complicated damage such as articular cartilage lesions, articular process fractures, or spinal cord lesions [5, 15]. As for the mechanism of onset in our patient, since he was involved in a head-on collision while driving and sustained a lacerated wound in the right inferior mandible, he presumably hit the right inferior mandible on the steering wheel, at which time excessive extension and rotation forces were applied to the cervical spine, resulting in rupture of articular capsule of C1-C2, leading to atlantoaxial rotatory fixation. The clinical findings showed typical torticollis after trauma and no features of neurological deficit.

In our review of 14 cases, 11 patients showed Fielding type I AARF and were treated with traction, manual reduction, or immobilization by cervical collar or halo vest, while the other 3 patients underwent surgery [5, 6, 8]. Most patients with Fielding type I undergo reduction using the traction method and immobilization with either a halo vest or different types of collars. Venkatesan et al. [13] reported 2 cases of AARF and commented that early recognition of atlantoaxial rotatory subluxation or dislocation is essential to successfully achieve closed reduction. Weißkopf et al. [16] pointed out that the success rate of conservative treatment decreases in proportion to the length of the dislocation treatment interval. Surgical stabilization is advised for cases of AARF showing spinal instability, neurological deficit, delayed diagnosis, failed reduction, and/or recurrent dislocation [1, 5, 6, 14].

Our patient presented with a very acute case of Fielding type I AARF with neither neurological symptoms nor complicating injuries such as bone fractures. We therefore performed manual reduction with the patient awake, and cervical pain was alleviated immediately after reduction. No previous reports have described awake manual reduction without the need for anesthesia, but this kind of manual reduction should be performed as soon as possible by experienced surgeons or by the right surgeons in the right places.

## Figures and Tables

**Table 1:** Reported cases with adult atlantoaxial rotatory fixation in the literature.

Author	Age and sex	Mechanism	Fielding type	Associated injuries	Duration to diagnosis	Reduction method and treatment	Results
Castel et al., 2001 [4]	41 M	Rugby injury	I	(—)	1 month	Reduction and Minerva jacket	Good

Fuentes et al., 2001 [5]	24 M	Suicidal jump	IV	Initial odontoid fracture only	1 month	C1-C2 fusion	Unknown

Kim et al., 2007 [8]	34 M	Fall down from high place	II	Lt superior facet fracture of C2	1 day	C1-C2 fusion	Good

Sinigaglia et al., 2008 [11]	26 F	Road traffic accident	I	(—)	45 days	Reduction and halo vest	Fair; because of cervical stiffness and headache
	21 F	Road traffic accident	I	(—)	1 day	Reduction and halo vest	Good
	29 M	Road traffic accident	I	(—)	1 day	Reduction and rigid collar	Good

Wang et al., 2008 [14]	44 F	Not described	I	(—)	6 months	Immobilization with halo vest	Poor; because of bilateral hand numbness

Goel et al., 2010 [6]	28 M	Fall down from high place	Not described	Odontoid fracture	1 day	Intraoperative facet manipulation	Good

Singh et al., 2009 [10]	25 F	Road traffic accident	I	(—)	0 day	Skull traction and halo brace	Good

Jeon et al., 2009 [7]	25 F	Road traffic accident	I	Thoracic fractures, alar ligament injury	5 days	Immobilization with Philadelphia brace	Good

Stenson, 2011 [12]	31 F	Falling backward	I	(—)	0 day	Immobilization with hard collar	Good

Marti et al., 2011 [9]	24 F	Stretching neck herself	I	(—)	1 day	Reduction and halo vest	Good

Venkatesan et al., 2012 [13]	20 F	Road traffic accident	I	(—)	0 day	Skull traction and hard collar	Fair; because of occipital pain
	52 F	Road traffic accident	I	(—)	0 day	Halo traction and hard collar	Fair; because of occipital pain
